# The Affective Core of the Self: A Neuro-Archetypical Perspective on the Foundations of Human (and Animal) Subjectivity

**DOI:** 10.3389/fpsyg.2017.01424

**Published:** 2017-09-01

**Authors:** Antonio Alcaro, Stefano Carta, Jaak Panksepp

**Affiliations:** ^1^Santa Lucia Foundation, European Centre for Brain Research Rome, Italy; ^2^Associazione Italiana Gestalt Analitica (AIGA) Rome, Italy; ^3^Department of Pedagogy, Psychology, and Philosophy, University of Cagliari Cagliari, Italy; ^4^Department of Integrative Physiology and Neuroscience, College of Veterinary Medicine, Washington State University, Pullman WA, United States

**Keywords:** self, affect, emotion, archetype, consciousness, intentionality, subcortical midline structures (SCMSs), neurodynamic patterns

## Abstract

Psychologists usually considered the “Self” as an object of experience appearing when the individual perceives its existence within the conscious field. In accordance with such a view, the self-representing capacity of the human mind has been related to corticolimbic learning processes taking place within individual development. On the other hand, Carl Gustav Jung considered the Self as the core of our personality, in its conscious and unconscious aspects, as well as in its actual and potential forms. According to Jung, the Self originates from an inborn dynamic structure integrating the essential drives of our “brain–mind,” and leading both to instinctual behavioral actions and to archetypal psychological experiences. Interestingly, recent neuroethological studies indicate that our subjective identity rests on ancient neuropsychic processes that humans share with other animals as part of their inborn constitutional repertoire. Indeed, brain activity within subcortical midline structures (SCMSs) is intrinsically related to the emergence of prototypical affective states, that not only influence our behavior in a flexible way, but alter our conscious field, giving rise to specific feelings or moods, which constitute the first form of self-orientation in the world. Moreover, such affective dynamics play a central role in the organization of individual personality and in the evolution of all other (more sophisticated) psychological functions. Therefore, on the base of the convergence between contemporary cutting-edge scientific research and some psychological intuitions of Jung, we intend here to explore the first neuroevolutional layer of human mind, that we call the affective core of the Self.

“I have long thought that, if there is any analogy between psychic and physiological processes, the organizing system of the brain must lie subcortically on the brain stem. This conjecture arose out of considering the psychology of an archetype [the Self] of central importance and universal distribution represented in mandala symbols. … The reason that lead me to conjecture a localization of a physiological basis for this archetype in the brain stem was the psychological fact that besides being specifically characterized by the ordering and orienting role, its uniting properties are predominantly affective. I would conjecture that such a subcortical system might somehow reflect characteristic of the archetypal form of the unconscious.”

Jung (1958, para. 582).

## Introduction

In the Western philosophic and scientific tradition, mental subjective life has been generally considered a prerogative of human beings that emerges from the activity of highly evolved and sophisticated neocortical cognitive functions. These views are sustained by the popular neurocognitive paradigm that is still deployed to localize subjective feelings within higher limbic and cortical brain regions (LeDoux, 1996; Rolls, 1999; Kandel, 2005), promoting the belief that consciousness only emerges from within the thalamocortical networks (Edelman, 1989; Crick, 1994). In accordance with such perspectives, psychoanalysts and cognitive psychologists considered that subjectivity is acquired during human individual development and derives from the introjection of some attachment-related operative models resulting in a kind of reflexive self-perception (Stern, 1985; Schore, 1994; Fonagy, 2001).

A strong version of such corticocognitive anthropocentrism has been recently re-affirmed by Joseph LeDoux in some publications (LeDoux, 2015; LeDoux and Brown, 2017). He considers human subjective experience as the result of higher-order cortical processes involved in the cognitive interpretations of our being in the world. The limitation of such a perspective derives from the erroneous idea that having a subjective experience necessarily corresponds to the fact of being self-aware of such an experience, as it can be tested asking “what do you feel?” to a human person. On the contrary, many different researchers in the field of psychology, philosophy and neuroscience have recognized the existence of subjective experiences without self-awareness, which presumably comes only as a secondary evolutionary product of our mental life (James, 1890/1950; Edelman, 1992; Searle, 1992; Damasio, 2010).

In line with the empiricist perspective of James (1890/1950), the neurophilosopher Georg Northoff defined the “phenomenal minimal Self” as the pre-reflexive form of subjectivity that presupposes an experience, defined by certain (pre)conscious qualities, or *qualia*, and the implicit sense of being part of such experience (Northoff, 2013; Northoff et al., 2014). Such a definition has the advantage of highlighting the two necessary and sufficient features of the Self: intentionality and conscious sensitivity (McGinn, 1989; Searle, 1991). Intentionality, as a goal-directed organized process, may be equated to a description of the Self as a dynamic patterning process unfolding toward a goal. Consciousness is the process through which the flux of dynamic changes pervading the Self is accompanied by qualitative phenomenal experiences.

Empirical human data indicate that the “minimal Self” is related to mental imaging and representation processing that emerge from “resting-state activity” of the CMS (Qin and Northoff, 2011; Musholt, 2013; Northoff et al., 2014; Hu et al., 2016; Weiler et al., 2016; LeDoux and Brown, 2017). Moreover, the CMS are anatomically and functionally closely connected with a set of SCMSs (Northoff and Panksepp, 2008; Panksepp and Northoff, 2009), that have been considered the neurophysiological substrate of an ancestral form of subjectivity, named the “Core-Self” by a member of this research group (Panksepp, 1998b) and the “proto-Self” by Damasio (1999). Interestingly, the SCMS are the most emotional parts of our brains (perhaps even more than the overemphasized limbic system), and the affective neurodynamics originating within the SCMS play a central role in the organization of human personality (Panksepp, 1998b, 2007; Davis et al., 2003; Northoff et al., 2006; Davis and Panksepp, 2011; Panksepp and Davis, 2014; Montag et al., 2016). Therefore, as direct expression of internal modes of functioning —of “intentions-in-action” (Panksepp, 1998b)—affects may be viewed as the basic organizers of the Self (Panksepp, 1998a,b, 2005, 2010, 2011; Damasio, 1999; Denton, 2006), and express a form of rudimentary consciousness (or proto-consciousness) that is characterized by primary-process and objectless (anoetic) feelings (Alcaro and Panksepp, 2014).

**FIGURE 1:**
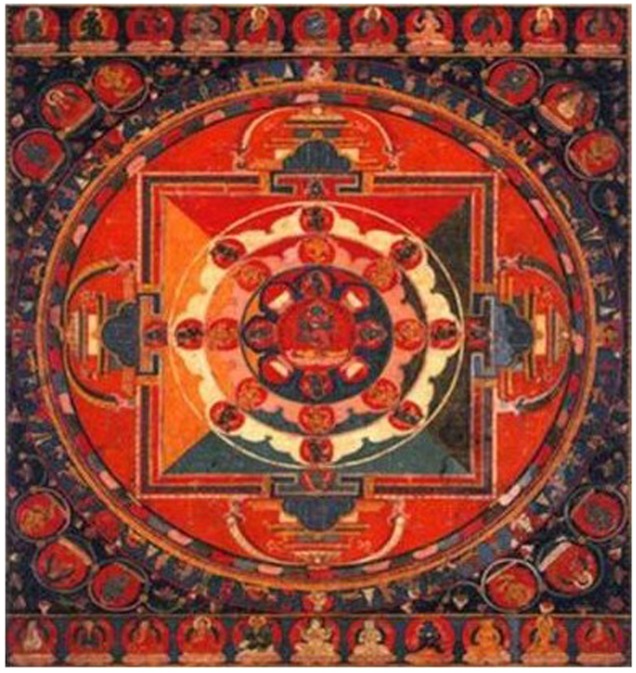
An example of Mandala.

At the beginning of this article we quoted a passage from Jung that contains three principal assertions, more or less explicitly expressed:

(1) Among all other Jungian archetypes^1^ there is one (the Self) that has a central importance and universal distribution and that is represented in highly organized geometric (and symbolic) “mandalic” patterns (Jung, 1933/1950, 1951) (**Figure 1**).

(2) If there is any correspondence between the brain and the psyche, the physiological bases of the Self might be localized in the brain stem, which is the foundational organizing system of the whole brain and mind.
(3) Besides its ordering and orienting role, the uniting properties of the Self are predominantly affective.

Accordingly, we think that such assertions by Jung were not only quite farsighted, but they actually opens ways to connect his theory of the psyche with the most advanced scientific theories and discoveries of our day.

## The Subcortical Core of the Self

**FIGURE 2:**
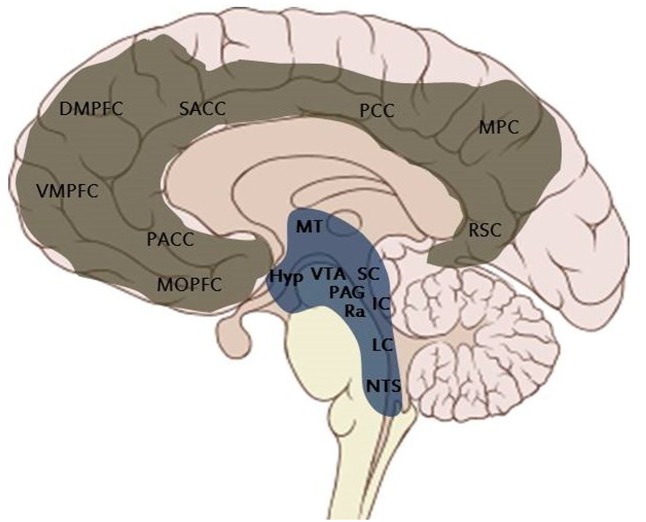
Schematic illustration of the Midline Structures of the Brain. Subcortical midline structures (in blue): Hyp, hypothalamus; IC, inferior colliculus; LC, locus coeruleus; MT, mediodorsal thalmus; NTS, nucleus tractus solitaries; PAG, periacqueductal gray; Ra, raphe nuclei; SC, superior colliculus; VTA, ventral tegmental area. Cortical midline structures (in gray): DMPFC, dorsal medial prefrontal cortex; MOPFC, medial orbital prefrontal cortex; MPC, medial parietal cortex; PACC, pre- and subgenual anterior cingulate cortex; PCC, posterior cingulate cortex; RSC, retrosplenial cortex; SACC, supragenual anterior cingulate cortex; VMPFC, ventral medial prefrontal cortex.

Human brain hemispheres are connected to the spinal cord and to the rest of the body through a set of SCMSs, whose functional anatomy is approaching maturity at birth (**Figure 2**). In his pioneering neuroevolutionary contributions, MacLean (1990) defines SCMS as the “reptilian brain,” since they are already present in reptiles and are conserved quite homologous (and not completely unchanged), in a stratified form, from birds to mammals. Although that metaphor has received considerable criticism, we envision that metaphor to mean that it is a “primary process” shared homologously (but with species-typical variations) by all vertebrates.

Human and animal studies show that SCMS lesions induce brain coma and the cessation of any form of psychic and intentional life: all mental activities collapse and organisms become zombie-like, exhibiting largely a vegetative existence without intentionality (Panksepp, 1998a,b; Watt and Pincus, 2004; Merker, 2007; Panksepp and Biven, 2012). Moreover, deficits get more severe the lower the damage occurs within the neuroaxis. For example, experimental studies on animals indicate that the complete destruction of the periaqueductal gray matter (PAG), which lies at the heart of the SCMSs, results in the destruction of all self-related processing of environmental events. With total damage to the PAG, all world-directed activities are compromised. Animals are marginally awake, but they do not appear to be conscious of things in any meaningful way (Panksepp and Biven, 2012, p. 409).

On the other hand, extensive lesions of the cortical mantle and higher limbic lobes do not destroy the field of consciousness For example, animals that have been neo-decorticated early in life sustain a remarkable level of behavioral coherence, intentionality and spontaneity. Not only do they show the ability to learn from positive or negative reinforced events, but they are actually more emotional than animal with intact brains (Huston and Borbely, 1973, 1974).

Concordant human evidence is available from a condition called hydranencephaly, in which cerebral cortex and higher limbic areas are totally destroyed *in utero*, which leaves most subcortical networks functional. Surprisingly, these hydranencephalic children express many signs of positive and negative affective states. For example, they:

“[…] express pleasure by smiling and laughter, and aversion by ‘fussing’ arching of the back and crying (in many gradations, their faces being animated by these emotional states). A familiar adult can employ this responsiveness to build up play sequences predictably progressing from smiling, through giggling, to laughter and great excitement on the part of the child” (Merker, 2007, p.79).

Moreover, they also show some forms of affective learning and memory. They:

“[…] take behavioral initiatives within the severe limitations of their motor disabilities, in the form of instrumental behaviors such as making noise by kicking trinkets hanging in a special frame constructed for the purpose (‘little room’), or activating favorite toys by switches, presumably based upon associative learning of the connection between actions and their effects. Such behaviors are accompanied by situationally appropriate signs of pleasure and excitement on the part of the child” (Merker, 2007).

In sum, animal and human data demonstrate that a primal form of Self emerges within SCMS and that all other sophisticated forms of mental life may depend on those brain regions, *since lesions or disturbances of the SCMS cause the collapse of any conscious and/or intentional activity*. In spite of the fact that this structure has been called the “core-Self” by a member of this research group (Panksepp, 1998b) and “proto-Self” by Damasio (1999), here we prefer to adopt the definition of “*affective core-Self*,” in order to underline the absolute relevance of the affective dimension. Indeed, as a consequence of its intrinsic neurophysiology and neuroanatomical organization, the SCMS are involved in the emergence of core affective states that have been differentiated into three categories (Panksepp and Biven, 2012):

(1) Homeostatic/visceral affects refer to internal bodily states that are perceived at a conscious level in the form of basic mood and feelings. Indeed, SCMS receive direct information from the internal body and in turn regulate visceral and somatic states, controlling the endocrine, the exocrine and the autonomous nervous systems^2^ (Damasio, 1996, 1999; Porges, 2011). Due to the strict connection with the “internal milieu” of the body (Bernard, 1865/1961), the SCMS have also been called the “visceral brain” (MacLean, 1990).
(2) Instinctual/emotional affects refer to intrinsic and highly valued dispositions to act, approaching or avoiding certain situations (see next paragraph). Indeed, SCMS are already evolutionarily furbished with neural systems that trigger and control instinctual action patterns and postures essential for organism survival and reproduction (such as locomotor and orienting movements, sexual behaviors, ingestive behaviors, etc.) (Tinbergen, 1951; Lorenz, 1965; Panksepp, 1998b; Denton, 2006; Arminjon et al., 2010). Some of such instinctual patterns consist of basic emotional dispositions, such as fear or rage or joy, etc. (Panksepp, 1998b) (see next paragraph).
(3) Sensorial affects refer to rudimental perceptual experiences with an intrinsic affective value, such as the feeling of touch, the hearing of calming or startling sounds, the sense of warmth or coldness, sweet or bitter tastes, and so forth. Indeed, SCMS are provided by some rudimental sensory way-stations for exteroceptive and proprioceptive perceptions (Merker, 2007). Interestingly, such subcortical perceptual areas are closely connected with neural motor nuclei responsible for directing attention, such as the superior culliculus and/or the optic tectum that control eye movements (Stein et al., 2002).

## Emotional Affects

**FIGURE 3:**
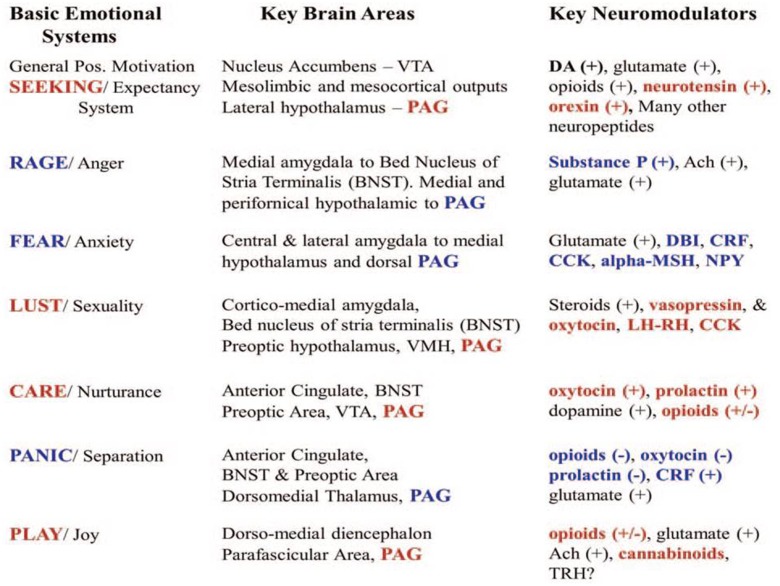
List of the Basic Emotional Systems with their neuroanatomy and neurochemistry. Red color refers to positive-appetitive emotional dispositions, while the blue color refers to negative-aversive emotional dispositions. Ach, achetilcholine; alpha-MSH, alpha-melanocyte stimulating hormone; BNST (bed nucleus of the stria terminalis); CCK, cholecystokinin; CRF, corticotropin releasing factor; DA, dopamine; DBI, diazepam binding inhibitor; LH-RH, luteinizing hormone-releasing hormone; NPY, neuropeptide Y; PAG, periacqueductal gray; TRH, thyrotropin-releasing hormone; VTA, ventral tegmental area.

Electric brain stimulation studies^3^ allowed localization within the SCMS of diverse mammalian Brain Operational Systems whose activation is responsible for the emergence of at least seven basic emotional dispositions (Panksepp, 1998a,b, 2005, 2010; Panksepp and Biven, 2012): the SEEKING System, the RAGE System, the FEAR System, the LUST System, the CARE System, the PANIC/Separation distress System and the PLAY System (**Figure 3**). Four of such Emotional Systems have been also found in the brain of reptiles, while perhaps lacking certain (or only marginally developed) more recently evolved social emotions (CARE, PANIC/Grief, and PLAY).

Emotional Operational Systems are instinctual neural devices releasing a series of coordinated and integrated responses that constitute the characteristic repertoire of an emotion^4^. Moreover, animal and human data strongly support the idea that the activation of each Emotional System modifies the organism’s subjective state and leads to characteristic feelings, which are perceived at a conscious or preconscious level. Indeed, human subjects report intense emotional feelings during the electric stimulation of the SCMS (Heath, 1964, 1996); while in animals such stimulations always induce rewarding or aversive effects that presumably will influence their behaviors in the future (Panksepp, 2010, 2015).

The (pre)conscious qualities of emotions are intrinsically related to their intentional character, since positive and negative affective feelings always reflect the intention to approach or avoid certain situations, allowing conceptualization of emotions as primal forms of intentional dispositions. For example, the SEEKING disposition drives the organism to forage for essential resources (food, water, sex, etc.), the FEAR disposition to avoid a source of danger, etc.^5^. Therefore, emotions always express a dispositional-intentional nature that projects the organism into the affordances of the world, thereby defining an animal’s key affective attitudes and, in conjunction with the cortical networks, scopes of behavioral strategies. This teleological view, fundamental also in Jung’s model of the psyche, considers emotional affects as autoperceptions of internal modes of functioning – of “intentions-in-action” (Panksepp, 1998b) – expressing a form of “anoetic” consciousness, which is the first primal layer of the brain in which the core-Self affectively experiences its own sense of itself^6^.

**FIGURE 4:**
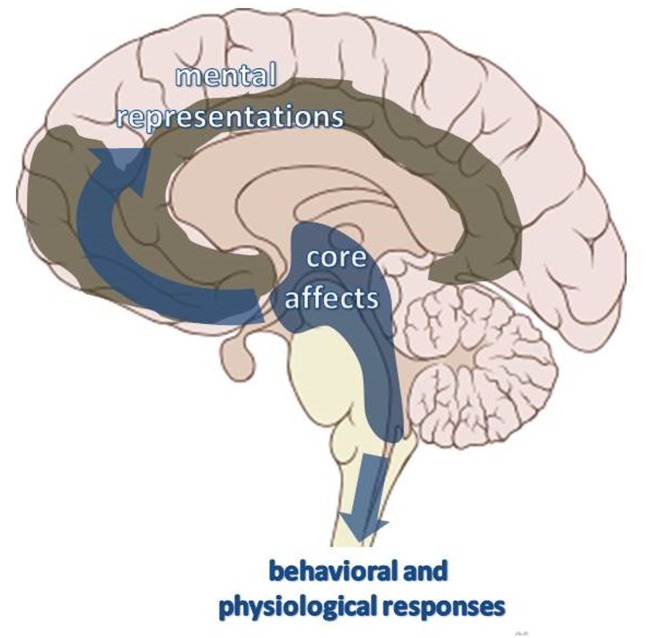
Schematic illustration of the ascending and the descending ways where core affective neurodynamic states originating within SCMS are transmitted.

In line with recent non-linear dynamic theories (Freeman, 1999; Brown, 2002; Llinas, 2002), emotional affects may be more properly identified with neurodynamic patterns that emerge within SCMS and then all along the brain and body, exerting an attraction over the organism’s activity and driving it toward specific “basins” of neuronal activation (see also Panksepp, 2000; Krieger, 2014). In such a way, emotional affects act as vectors that orient the ensemble of behavioral and mental activities toward specific directions and orbits of meaning. While being transmitted toward the spinal cord and other motor or visceral effector systems, they become integrated ensembles of physiologic and behavioral instinctual responses. In diffusing toward higher brain areas, they can take the form of ensembles of mental representations (archetypal images or thoughts), gravitating around a characteristic affective core (**Figure 4**).

When neurodynamic patterns evolve in a rigid and automatic way, they give rise to stereotyped behavioral or mental actions that are characterized by compulsiveness and act on the subjective world as external factors, or to use a Jungian terminology, as “ectopsychic factors”:

“Among the psychological factors determining human behavior, the instincts are the chief motivating forces of psychic events. […] if we look upon the appearance of the psyche as a relatively recent event in evolutionary history, and assume that the psychic function is a phenomenon accompanying a nervous system which in some way or another has become centralized, then it would be difficult to believe that the instincts were originally psychic in nature. And since the connection of the psyche with the brain is a more probable conjecture than the psychic nature of life in general, I regard the characteristic *compulsiveness* of the instinct as an ectopsychic^7^ factor. None the less, it is psychologically important because it leads to the formation of structures or patterns which may be regarded as determinants of the human behavior” (Jung, 1937/1942, para. 234).

However, emotional dispositions differ from rigid behavioral automatisms, since the ensemble of actions coordinated by each Emotional System is organized and modulated in a flexible way. As a consequence of that, emotions have been conceptualized as “flexible action patterns” (Llinas, 2002) which respond to trigger stimuli, but also *anticipate* future events, *prepare* the organism to cope with uncertain situations and with sufficient neocortex, to *orient* its attitude toward specific intentional routes. The intrinsic non-deterministic quality of emotional dispositional patterns opens the material/organic structure to the entrance of a psychic intentional/conscious germ (Bergson, 1896/1991). As underlined by Jung:

“[…] the immediate determining factor is not the ectoplastic instinct but the structure resulting from the interaction of instinct and the psychic situation of the moment. The determining factor would thus be a *modified* instinct. The change undergone by the instinct is as significant as the difference between the color we see and the objective wave-length producing it. Instinct as an ectopsychic factor would play the role of a stimulus merely, while instinct as a psychic phenomenon would be an assimilation of this stimulus to a pre-existing pattern. A name is needed for this process. I should term it *psychicization*^8^” (Jung, 1937/1942, para. 234).

## Core Affects as Archetypal “Psychoid” States and the Dual-Aspect Monism

At the current historical moment, scientific research is not able to explain how SCMS neurodynamic activity is related to the emergence of core affective states, that are experienced at the subjective level (consciousness) and that may teleologically influence the course of material events (intentionality) (Jonas, 1976; Searle, 1992). This difficulty probably arises when subjective states (first-person experiences) are reductionistically derived from objective material processes (third-person events), in the erroneous attempt to restrict an intensive and non-localizable phenomenon within a three-dimensional spatial field (Bergson, 1896/1991).

A very influential empirically oriented philosophic tradition considers affective feeling as intrinsically related to the perception of internal visceral and bodily states, a kind of integrated enteroception. The most famous theory within this perspective was advanced independently by James (1884) and Lange (1887). They suggested that affective feelings reflect cortical-cognitive representation of peripheral-unconscious arousal activated within the body by certain instinctual/emotional devices. Recently, Antonio Damasio proposed a new version of the James-Lange theory, correcting its original cortico-centrism and suggesting that the SCMS are involved in a first-step representation of somatic and visceral states (Damasio, 1996, 1999, 2010).

Damasio’s conception has illustrious antecedents in the history of psychoanalysis. Indeed, considering affects as states of visceral tension and relaxation, Freud wrote:

“The Id, cut off from the external world, has a world of perception of its own. It detects with extraordinary acuteness certain changes in its interior, especially oscillations in the tension of its instinctual needs, and these changes become conscious as feelings in the pleasure-unpleasure series” (Freud, 1940/1964, p. 190).

The Freudian visceral view has been recently taken again by neuro-psychoanalists, who postulate a strict correspondence between the internal physiologic environment and the mental world of the subjects (Solms and Turnbull, 2002). In such a perspective, affective feelings are linked to the process of homeostasis (Cannon, 1929; Denton, 2006), and then to the messages of survival and reproductive success or failure that the body sends to the brain–mind. Moreover, recent developments within the neuropsychoanalytic homeostatic perspective has also suggested that embodied interactions with other people in childhood permit the “mentalization” of basic visceral sensations, transforming rough internal perceptions into subjective feelings (Fotopoulou and Tsakiris, 2017).

However, the physiological connection of SCMS with the organism’s internal and external environment does not imply that affective feelings simply originate from neurocognitive representations of visceral and somatic functions: for instance, reflecting about the nature of affects, Freud himself affirmed that he was skeptical that:

“With this enumeration we have arrived at the essence of an affect. We seem to see deeper in the case of some affects and to recognize that the core […] is the *repetition of some particular significant experience*. This experience could only be a *very early impression of a very general nature, placed in the prehistory not of the individual but of the species”* (Freud, [28], p. 395).

In this highly significant passage, Freud abandoned his traditional reductionist perspective to embrace a Lamarkian and anti-reductionist approach^9^. According to him, the intrinsic core of an affect is not just a somatic material process, but a psychic event (“a very early impression of a very general nature, placed in the prehistory not of the individual but of the species”). Therefore, the reductionist stand must not exclude the more encompassing, non-reductive paradigm that Jung constantly developed in his writings. Perhaps, the best example of this double vision – reductive and non-reductive – may be found in the essay “On the nature of the psyche” (Jung, 1947/1954), where he takes into account both biological components (for instance the instinctual “patterns of behavior” in animals and man), and their relationship to non-causal principles related to subatomic physics.

This issue leads us directly to touch upon a philosophic position known as “*dual-aspect monism*” adopted first by Spinoza (see Ravven, 2013) as well as by Jung and Pauli many years ago (Atmanspacher, 2012), and recently re-proposed by Mark Solms and other neuro-psychoanalysts (Kaplan-Solms and Solms, 2000). According to such a view, the material and the subjective worlds are two complementary manifestations of a unique, albeit perhaps unknowable unitary reality, to which Jung refers with the concept of “psychoid.” The presence of such underlying dimension has been widely underlined in Eastern cultural tradition, as well in some Western philosopher, such as A.N. Withehead (1929). In the 20th century, its existence was revealed by the study of quanto-mechanic processes in physics, and of unconscious processes in analytical psychology^10^. Both disciplines recognized the influence of unobservable (paradoxical) phenomena within the normal flow of observable material and mental events.

Today, the application of non-linear dynamic theories to neuroscience (Freeman, 1999; Brown, 2002; Llinas, 2002; Krieger, 2014), shifting the level of neurological analysis from a material-neurochemical level to an immaterial-electrical field level, theoretically opens the way to shift from the physics of massive bodies to quantum physics, and, therefore, to the level of reality in which synchronistic phenomena may be rationally admissible^11^ (Jung, 1955; Bohm, 1980/1981; Penrose, 1989; Brown, 2002). For example, the neurologist Brown (2002), who integrated the process philosophy of Whitehead with the most recent discoveries of neuroscience, looks at the Self as the microgentic oscillatory quantistic process that emerges within the brain from the depth (subcortical) to the surface (cortical), giving rise to moments of consciousness, that gradually evolve from pure arousal without objects to complex representations and self-representation^12^.

In line with such speculative hypotheses, we may represent *core affects* as “archetypes-as-such,” primal organized configurations of intrinsically evaluative events that reveal themselves both in brain-behavioral action/dispositional patterns (objective domain) as well as in intense affective feelings (subjective domain). Such *archetypal psychoid* events presumably correspond, at a physical descriptive level, to a dynamic structure of global-field quantistic microwaves reverberating within SCMS^13^ (see Brown, 2002). In amplifying quantistic microwaves (Penrose, 1989; Brown, 2002), the brain may be more properly viewed as a transformer station, connecting mind and matter, and actualizing psychological processes within a linear spatio-temporal dimension. As suggested by Jung:

“One might assume the psyche gradually rising from minute extensity to infinite intensity, transcending for instance the velocity of light and thus irrealizing the body. […] In the light of this view the brain might be a transformer station, in which the relatively infinite tension or intensity of the psyche proper is transformed into perceptible frequencies or “extensions.” Conversely, the fading of introspective perception of the body explains itself as due to a gradual “psychification,” i.e., intensification at the expense of extension.

Psyche = highest intensity in the smallest space. UNEXTENDED INTENSITY^14^”

(C.G. Jung ∼Carl Jung, Letters Vol. II, PP 43–47).

## Affects as the Primal Organizers of the Subjective Life

Recent neuroscientific formulations affirm that affectivity is the primordial form of subjectivity. More specifically, it has been recognized that affects express an enlarged, diffused and primary-process sensitivity devoid of any specific content or clear cognitive distinction between the external-objective and the internal-subjective world. Such primary-process and objectless sensitivity has also been called “*anoetic*” consciousness (or protoconsciousness) an “[…] unthinking form of experience, which may be affectively intense without being known” (Solms and Panksepp, 2012, p. 149)^15^. It is “the rudimentary state of autonomic awareness […], with a fundamental form of first-person ‘self-experience’ which relies on affective experiential states and raw sensory and perceptual mental existences” (Vandekerckhove and Panksepp, 2009, p. 1). Within this context, anoetic proto-conscious feelings emerge as neurodynamic “wave’s crest” from a continuous flux of diffuse arousal without focus or intent (mood)^16^.

As a matter of fact, affectivity was considered to be the fundamental feature of the brain–mind by many early pioneers, including John Hughlings Jackson, and also Eugen Bleuler, one of the most important partners of Jung’s scientific career. Here, is what Bleuler wrote in his seminal work on psychosis:

“[…] the little child has a fully developed affectivity; all the affects present in the adult are wholly developed in him. On the contrary, the child’s intelligence is void of contents and the logical processes are relatively poor” (Bleuler, 1906/1912, p. 24, our translation).

Also Jung, from the very beginning – i.e., from his *Association studies* (Jung, 1904/1905) – gave an absolute relevance to affects and affective feelings for subjective mental life, as underlined in the following passage:

“Every psychic process has a value quality attached to it, namely its feeling-tone. This indicates the degree to which the subject is affected by the process or how much it means to him (in so far as the process reaches consciousness at all). It is through the ‘affect’ that the subject becomes involved and so comes to feel the whole weight of reality” (Jung, 1959/1978, para. 61).

Therefore, Jung considered affects as forces that attract the subject and that are experienced in the form of characteristic feelings. In this sense, Jung (1928/1948) substituted Freud’s theory of the impulse (the drive) with a much more cogent theory of a binding field-force (in psychological terms, the affect) which polarizes and confers value on patterns, or psychic representations.

Therefore, affects may be viewed as transpersonal forms of experience pervading a primal subjective field, that is not yet individually conscious (or self-conscious), since it lives within an undifferentiated organism/environment continuum. At the same time, affects are the bridge between the collective mind and the individual mind, between the instinctual inheritance of the specie and the personal experiences accumulated within the individual life. As described by member of this research group:

“By providing a shared neural platform for diverse affective experience, the core Self can be considered a nomothetic (universal) brain function. As the core Self, along with the many raw feelings it elaborates, interact with higher cognitive […] processes, it promote the emergence of various ideographic (individually unique, experientially refined) extended selves, during development brain maturation” (Panksepp and Biven, 2012, p. 392).

Although affects are originally objectless experiences, they provide the field whereby all incoming perceptual stimuli/experiences are integrated into a single conscious and intentional state. Moreover, affective states exert a powerful modulation over every form of individual learning and associative memory (learning modulation property) (Panksepp and Biven, 2012; Vandekerckhove et al., 2014). Thereby, all personal experiences that are accumulated during individual history take the form of clusters (or complexes) of perceptual memory traces gravitating around an affect. This is precisely the idea developed by Jung (1960) with the concept of “feeling-toned complexes,” which he considered the psychological structures that gather together different mental contents and representations on the base of a common affective state^17^. Each complex is united by the same emotion, which define its core of meaning, and organizes experience, perception, and affect around a constant central theme. For example, the complex of inferiority is a constellation of memories, thoughts and phantasies related to the lack of self-worth, a doubt and uncertainty about oneself, and feelings of not measuring up to standards.

There, speaking of the complex, Jung wrote: “the constellating power of its nuclear element corresponds to *its value intensity*, i.e., to its energy” (Jung, 1928/1948, §19, Jung’s italics). Along these lines, he also wrote: “It would be an unpardonable sin of omission were we to overlook the *feeling-value of the archetype*. This is extremely important both theoretically and therapeutically” [CW 8, para. 411], since the archetype is a “[…] living system of reactions and aptitudes” connected with the living individual “[…] by the bridge of emotion” [CW 18, para. 589].

The Jungian theory of the feeling-toned complexes is an elaboration of the work of Pierre Janet on the autonomous fixed ideas. According to Janet (1889), fixed ideas are mental images or thoughts that have a high emotional charge and take on exaggerated proportions, so they may not be normally integrated within the ego-consciousness and become isolated from the habitual personality, creating dissociated states of the mind (Monahan, 2009). However, in contrast with the original theory of Janet, Jung sustained that the dissociative aspect of complexes is usually reversible, so they may be much or more integrated according to the momentary situation. Only in severe mental pathologies, such as psychoses, certain complexes are permanently dissociated from the conscious ego and the personality becomes fragmented.

## A Multilayered Neuro-Evolutive Architecture of the Self

Many authors had hypothesized that the Self is not a unitary phenomenon and that it may be differentiated in overlapping layers that have been stratified in evolution (James, 1890/1950). The use of an archeological metaphor to describe the human mind has been widely used by psychoanalysts. For example, Sigmund Freud looked at the psyche as an ancient town, like Rome, where the passage of time has been impressed into a monumental stratification that goes from antiquity to the modern era. The exploration of the multilayered organization of the human mind has been empirically enriched by contributions coming from the neurosciences. John Hughlings Jackson, the father of English neurology, was probably the first neurophysiologist offering an explicit hierarchical description of brain functioning (see Franz and Gillett, 2011), and his interesting intuitions were further developed by Lurija (1962). Moreover, integrating human and animal neuroanatomic perspectives, MacLean (1990) gave an essential neuroevolutionary view to modern neuroscience (albeit, not without abundant debate – for overview, see, Panksepp, 2002). He conceptualized three overlapping layers in the human brain: the neocortical-rational brain, characteristic of our species, an intermediate limbic-emotional brain, characteristic of all mammals, and the visceral-instinctual brain, characteristic of reptiles—a pattern that is more or less conserved across vertebrates^18^.

However, in contrast to dominant anthropocentric perspective, accumulating neuroscientific evidence affirms that a primordial form of subjectivity is already present at the first level of the brain hierarchy (the reptilian-paleomammalian instinctual brain) and that it is widely diffused across mammals, birds, and perhaps other vertebrates (Panksepp, 1998b, 2016; Seth et al., 2005; Northoff and Panksepp, 2008; Edelman and Seth, 2009; Panksepp and Northoff, 2009; Revonsuo, 2010; Ward, 2011; Panksepp and Biven, 2012; Fabbro et al., 2015; Feinberg and Mallatt, 2016). The accruing evidence led a member of this research group to introduce some cardinal changes into the tripartite neuroevolutionary model of Paul Maclean^19^ (Panksepp, 1998b, 2011; Panksepp and Biven, 2012), and to coax the contemporary neuro-psychoanalytic movement to consider the reality of an affective (if not cognitively reflective) “conscious Id”^20^ (Solms and Panksepp, 2012; Solms, 2013).

In accordance with such hypotheses, it has been recently proposed that the phenomenon of consciousness may be differentiated into anoetic, noetic and autonoetic states, that correspond to different layers of the neuro-psychic architecture (Vandekerckhove and Panksepp, 2009, 2011).

Anoetic consciousness (already described in the previous paragraphs) is the first primal layer of the Self^21^, an ancient neuroevolutionary product whose existence is widely diffused across animal species, extending from mammals to birds and reptiles, and probably to vertebrates in general (Fabbro et al., 2015; Feinberg and Mallatt, 2016). Moreover, newborns come into the world with an inherited ability to experience anoetic states as the result of the activity of subcortical brain areas already functionally mature at birth (Merker, 2007), as we described within this article.

On the other hand, noetic and autonoetic consciousness are more recent evolutionary functions of the superior cortical and limbic areas of the brain and require some form of individual learning to be adequately developed and expressed.

Noetic consciousness reflects knowledge-based experiences that arise when refined attentional capacities permit a clear distinction and categorization of specific features of the environment which, with enough neo-cortex, allows animals to think ahead. Indeed, when specific aspects of events become the focus of attention, explicit object-related reflective awareness comes into the fore while semantic (conceptual) memory helps to analyze and categorize the situation (Tulving, 1985). This is the form of consciousness that Edelman called “primary consciousness,” and that he related to the activity of re-entrant thalamo-cortical brain circuitries (Edelman, 1989). Indirect evidence suggest that noetic consciousness is present across mammals, birds, and perhaps also in reptiles (Edelman et al., 2005), and that human babies, born very immature, start to manifest it after the third month of development.

A further step in the neuro-psychic evolution is accomplished through the acquisition of language (Hauser et al., 2002; Gazzaniga, 2011) and of the ability to mentally travel in time (Corballis, 2012), making possible not only the conscious recall of past events (episodic memory), but also to imagine future events including one’s own death (Tulving, 2002). This narrative structure of the Self, which presumably appeared more than 1 million years ago, most clearly (for us) in the genus *Homo habilis*, gives rise to the most sophisticated, self-awareness based, autonoetic consciousness, that Edelman has described as “secondary consciousness” (Edelman, 1992). Interestingly, autonoetic consciousness seems heavily related to the so-called “theory of mind” (the ability to imagine what’s going on the mind of other individuals), which seems to be a chiefy human feature (Corballis, 2012).

The passage from the affective core Self (anoetic consciousness) to higher stages of Self-evolution (noetic and autonoetic consciousness) is an evolutionary leap that humans achieved; a leap that has been long prepared by antecedent evolutionary processes in other mammals, perhaps birds, and other creatures, as well. In future contributions, we will explore and describe such evolutionary progressions, as well as the new creative potential opened up by the acquisition of cognitive reflexive abilities^22^. Here, we have focused on the most basic foundations of our being–the affective substrates from which all our higher psychological functions derive. These initial layers of our minds, provide an essential foundation for all subsequent higher-order psychoneurological functions that make human minds unique.

## Conclusion

Although dominant neurocognitive paradigms typically co-locate subjective life to the highest levels of the brain organization, primarily as the consequence of accumulating individual memories that are stored within neuroplastic forebrain circuits, a large amount of neuro-ethological evidence shows that non-human animals (mammals, birds, and perhaps also other vertebrates) also have forms of subjectivity that emerge from the activity of old evolutionary subcortical brainstem, diencephalic, and basal forebrain areas (Panksepp, 1998b, 2015, 2016; Seth et al., 2005; Edelman and Seth, 2009; Revonsuo, 2010; Ward, 2011; Panksepp and Biven, 2012; Fabbro et al., 2015). These findings clearly indicate that subjectivity is an inherited disposition routed on the instinctual archaic action-foundations of our brain (Goodwyn, 2010), and they confirm Jung’s view that *before* reflexive self-consciousness is developmentally acquired by infants, a primordial-instinctual affective form of Self already exists, expressing itself in the form of a affective-psychic intentionality that can interact effectively, in an evaluative way, with the material, deterministic world.

In his famous autobiography (Jung, 1962/1963), Carl G. Jung reported a personal dream that he considered the most important revelation about the structure of the psyche:

“I was in a house I did not know, which had two storys. *It was ‘my house*.’ I found myself in the upper story, where there was a kind of salon furnished with fine old pieces in rococo style. On the walls hung a number of precious old paintings. I wondered that this should be my house, and thought, ‘Not bad.’ But then itoccurred to me that I did not know what the lower floor looked like.

Descending the stairs, I reached the ground floor. There everything was much older. I released that this part of the house must date from about the 15th or the 16th century. The furnishings were medieval, the floors were of red brick. Everywhere it was rather dark. I went from one room to another thinking “now I really must explore the whole house.” I came upon a heavy door and opened it. Beyond it, I discovered a stone stairway that led down into a cellar.

Descending again I found myself in a beautifully vaulted room which looked exceedingly ancient. Examining the walls, I discovered layers of brick among the ordinary stone blocks, and chips of brick in the mortar. As soon as I saw this I knew that the walls dated from Roman times. My interest by now was intense. I looked more closely at the floor. It was of stone labs and in one of these I discovered a ring. When I pulled it the stone slab lifted and again I saw a stairway of narrow stone steps leading down into the depths.

These, too, I descended, and entered a low cave cut into the rock. Thick dust lay on the floor, and in the dust were scattered bones and broken pottery, like remains of a primitive culture. I discovered two human skulls, obviously very old and half disintegrated. Then I awoke” (Jung, 1962/1963, p. 155).

Such a dream is another example of how the psyche may be composed of multiple neural strata where more superficial and recent layers have been built upon the older ones during the natural (and cultural) history of our species. In thinking about the meaning of the deepest and primordial layer, the cave, Jung wrote that:

“[Here] we reach the naked bed-rock, and with it that prehistoric time when reindeer hunters fought for a bare and wretched existence against the elemental forces of wild nature. The men of that age were still in full possession of their animal instincts, without which life would have been impossible” (63, par. 55).

## Author Contributions

AA had the original idea and wrote the first draft of the manuscript. SC introduced important theoretical and clinical contribution, especially in relation to Jungian perspective. JP worked on the basic neuroscientific parts and revised the entire manuscript.

## Conflict of Interest Statement

The authors declare that the research was conducted in the absence of any commercial or financial relationships that could be construed as a potential conflict of interest.

## Footnotes

1 The best definition of an archetype is an “instinct of imagination,” that is an inherited disposition to imagine a cluster of representations gravitating around a characteristic meaning. The correspondences between the concept of archetype and that of instinct was repeatedly underlined by Jung and his successors (see Stevens, 2003). From the many descriptions of the archetype, we will quote the famous footnote in which Jung tries to distinguish between archetype-as-such and archetypical images: “By this I do not mean the existing form of the motif but its preconscious, invisible ‘ground plan.’ This might be compared to the crystal lattice that is preformed in the crystalline solution. It should not be confused with the variously structured axial system of the individual crystal” (Jung, 1928/1948, footnote para. 590).

2 The endocrine system refers to the collection of glands of an organism that secrete hormones directly into the circulatory system to be carried toward distant target organs. Exocrine glands are glands that produce and secrete substances onto an epithelial surface by way of a duct. Examples of exocrine glands include sweat, salivary, mammary, ceruminous, lacrimal, sebaceous, and mucous. The autonomic nervous system (ANS) is a division of the peripheral nervous system that influences the function of internal organs. The ANS is a control system that acts largely unconsciously and regulates bodily functions such as the heart rate, digestion, respiratory rate, pupillary response, urination, and sexual arousal.

3 Electric brain stimulation is an experimental technique that is generally used in animal studies, but that has been also utilized in humans (Heath, 1996). The insertion of a microelectrode in a specific brain site permits electric impulses to be sent that stimulate neural assemblies and circuits. Behavioral responses to such stimulations are observed in animals, while in humans is also possible to ask what is going on during the period of stimulation.

4 For example, the Rage System activates the sympathetic nervous system and a sequences of movements and postures that serve to attack and defend and that have also acquired a communicative meaning.

5 From a Darwinian point of view, each emotional disposition serves a specific role in terms of organism survival and/or reproduction. However, the immediate adaptive function of each emotion does not exhaust the ensemble of possibilities opened up by its expression. For example, the SEEKING disposition also drives animals to explore novel environments or contexts, and sustains creativity in pushing to search for novel solutions. Therefore, we may look at emotions as very general attitudes whose adaptive function constitutes a specification that natural selection crystallized during biological evolution.

6 Strictly speaking, this is not yet a representation (a *vorstellung*), it is a *presentation* (a *darstellung*) generated, in Jungian terms, by one of the five basic human “instincts”: reflectivity (Jung, 1937/1942) applied to the psychoid patterning process (which we may describe as analogous to the neural networks).

7 This means that the psychic characteristic of an instinct is an emergent quality of an originally not-psychic compulsive/automatic urge/pattern (such as that of an insect) that could activate the organism. This urge/pattern is wholly ectopsychic, as it originates not just *outside* the organism (the “outer” world), but *outside the psyche*, at a wholly physiological level. With the evolution of a centralized nervous system, this ectopsychic urge/pattern is modified by the nervous system and, so modified, becomes “psychicized,” i.e., it becomes the function of the interaction between the ectopsychic stimulus and the “inner” nervous system that processes it. Such an interaction is strictly connected to Jung’s four functions that organize consciousness, as they may be directed toward ectopsychic inputs (in extroversion) or endopsychic stimuli (in introversion).

8 If we apply those considerations to affective neuroscientific evidence, it seems reasonable to imagine that the “psychic situation of the moment” should somehow find a physical (i.e., something that belongs to physics) correspondence within a continuous flow of SCMS neurodynamic activity that presents some essential bio-physic character associated with subjective conscious states (Edelman and Tononi, 2000). When Emotional Systems are activated, they release an ensemble of neural, visceral, and motor patterns that reverberate within the SCMS and interact with pre-existing neurodynamic patterns, giving rise to a “modified instinct” which expresses a “psychicization” (or “subjectification”) factor.

9 It seems then that Freud and Jung, along with the many other scientists of their time, supported a mixed Darwinian/Lamarkian evolutionary paradigm, although in Jung’s views the role of random changes (e.g., mutations) was considered a weak construct not just in regard to evolutionary concerns, but especially, and more radically, as a foundational epistemological principle. Thus, in Jung’s thought, the Lamarkian perspective seems to be much more central than in Freud’s; as, for Jung, all complex systems (“nature”) develop following not only a evolutionary causal *push*, but also, and especially, a virtual teleological *pull*. And if one admits an evolutionary virtual *attractor* – a goal – the question whether such a goal may be irrational, may become an urgent concern for the generation of a decisive formal hypothesis. For Kauffman (1993) if evolutionary bifurcations and migrations of populations had to follow pure Darwinian chance, few emerging specialized phenotype would actually survive, but undergo extinction, as the mathematical model of such a purely Darwinian evolutionary pattern would cause what Kauffman calls catastrophe of complexity. This term describes the real outcome of such an evolutionary model based on pure chance: the “regression” of the more specialized species to some modal average as opposed to their stabilization at an evolutionary peak. For Kauffman, in order for evolution to maintain its peaks, as reached by the more adaptive species, one needs to assume a self-regulating emerging organizational order intrinsic to every non-linear complex system (such as a living organism). In our opinion, such self-organizing order represents another example of a virtual teleological factor within an evolutionary process (see also, Tagliagambe and Malinconico, 2011).

10 In a letter to Rosenfeld of April 1, 1952 (p. 593), Pauli writes: “For the invisible reality, of which we have small pieces of evidence in both quantum physics and the psychology of the unconscious, a symbolic psychophysical unitary language must ultimately be adequate, and this is the distant goal to which I actually aspire.” Meyenn, K. von (Edn 1996): Wolfgang Pauli. *Wissenschaftlicher Briefwechsel*, Band IV, Teil I: 1950/1952, Berlin: Springer.

11 Besides the work by Llinas and Freeman, which we already briefly noted, we would also like to recall Eccles’s (1994) observation that the number of molecules exchanged through the synaptic membrane is so small that it should not follow the statistical behavior of massive bodies, but that of quantistic entities, which would, in his opinion, support his and Karl Popper’s theory of a mind that can actually initiate various activities of the brain.

12 In the last 10 years, accumulating scientific evidences indicate that global-field quantistic microdynamic states, which have not yet encountered a collapse in their wave functions, may influence the behavior of animals and other living organisms (McFadden and Al-Khalili, 2014). The sensitivity that biological organisms show for non-local quantistic oscillations confirm the speculative hypotheses formulated by the pioneers of quantum physics, that tried to give a new scientific status to concepts like “soul,” or “psyche,” or “life” (Schrödinger, 1944; Heisenberg, 1958/2000). Interestingly, it has also been suggested that the affective dimension is the most direct manifestation of a pervasive quantistic field which influence animal’s behavior from within (Simondon, 1989/2007).

13 Their action has been evolutionarily impressed into the organisms and may be elicited by certain neural devices that have been conserved due to their adaptive value. When brain affective systems are stimulated, they activate highly sensitive neuro-physiologic states that may resonate with the quantic spatio-temporal vectors of core affects and with their subjective complementary qualities.

14 In light of the uncanny epistemic nature of the human mind, basically rooted in its ability to think natural numbers, the possibility that nature is trying to converge toward not survival but knowledge (the Greek “gnosis”) does not seem at all obsolete (but is perhaps ontologically obscure). In any event, it is precisely under this perspective that the very ultimate function of the brain (and therefore the body) may be re-thought in less neuro-reductionist and more encompassing psychological terms. Jung’s very far-fetched hypothesis quoted above is just such a speculative hypothesis.

15 In the clinical situation, these feelings remind us of Christopher Bollas’ description of the mood that belongs to what he calls the “unthought known,” and which he directly refers to the primary pre-objectual relationship with the “environmental mother” (Bollas, 1987).

16 Lacking any boundaries or subject/object differentiation, affects tend to be projected into the external world, coloring objects of perception by specific affective tonalities and intrinsic intentional aims (projection). This kind of projective attitude may at times create a strict synthesis (or overlapping) between affects and external objects, so the object is perceived to be carrying an affective value of its own. For example, when we are under the influence of our intrinsic affectiv e selves, we often do not recognize that we are loving or hating a person, but feel instead that such a person is lovable or hateful.

17 As an affective organizer, the “complex” is a Gestalt, or, in contemporary language, an example of a “dynamic system.” A strong support for this view may be found in Paul Weiss’ biological models of non-linear complex systems to describe the intrinsic organization of all living “systems” and its application in psychology by Sander (2007) and the so-called Boston group.

18 The neuroevolutionary layers of Paul Maclean (which has antecedents going back to Hughlings Jackson shows some correspondence with the tripartite organization of the psychic apparatus described by Sigmund Freud. Indeed, the subcortical reptilian brain may be equated to the Id, since it is the seat of instinctual needs (drives) that sends ascending reticular projections that modulate forebrain arousal states (Moruzzi and Magoun, 1949). On the other side, the Ego and the SuperEgo may be related to certain neuro-functional processes located within the upper limbic and neocortical regions, where interiorized object-relationships exert an inhibitory control over spontaneous drive’s expression (Carhart-Harris and Friston, 2010; Solms and Panksepp, 2012).

19 As in MacLean’s view, also for Panksepp the topmost and most recent layer is the neo-cortical brain, responsible for sophisticated cognitive functions, such as language and self-awareness. However, in Panksepp’s perspective, the second layer of the MacLean schema, the limbic brain, is mainly involved in emotional learning and higher regulatory processes, instead of in unconditional emotion generation and expression. Moreover, the most ancient and deep part of the brain, that MacLean indicated as the reptilian brain, is responsible not only for basic instinctual and homeostatic processes, but also for the emergence of primary-process, basic primordial emotions, with their characteristic affective feelings (which are further elaborated by the higher brain levels).

20 Despite the importance attributed to inherited instinctual dispositions shared by all vertebrate animals, it is noteworthy that Freud excluded that the possibility that the instinctual “Id” might have any kind of psychological attributes, such as intentionality or consciousness, that may be essential to integrate the ensembles of higher brain functions into coherent subjective states.

21 Strictly speaking, this form of consciousness is not is not yet based a representation (a *vorstellung*), it is a *presentation* (a *darstellung*) generated, in Jungian terms, by one of the five basic human “instincts”: reflectivity (Jung, 1937/1942) applied to the psychoid patterning process (which we may describe as analogous to the neural networks).

22 This leap, a second evolutionary step that we will discuss in a second article, is based on the fundamental process of self-reflectivity [one of Jung basic five “instincts” (Jung, 1937/1942)] of a pattern into an image. It is intrinsically connected to dreaming and, as already noted by Jouvet (1975), may be regarded as the self-representation of the psyche updating its strategy for survival, starting from its ancestral ectodermic (cold-blooded) repertoire of its reptilian past.

## Abbreviations

CMSs: cortical midline structures
SCMSs: subcortical midline structures.
